# Health status measured by Kansas City Cardiomyopathy Questionnaire-12 in primary prevention implantable cardioverter defibrillator patients with heart failure

**DOI:** 10.1186/s12872-021-02218-9

**Published:** 2021-08-28

**Authors:** Gustav Mattsson, Marita Wallhagen, Peter Magnusson

**Affiliations:** 1 Centre for Research and Development, Uppsala University/Region Gävleborg, 801 87 Gävle, Sweden; 2 Department of Building Engineering, Energy Systems and Sustainability Science, University of Gävle, 80176 Gävle, Sweden; 3 Cardiology Research Unit, Department of Medicine, Karolinska Institutet, 171 76 Stockholm, Sweden

**Keywords:** Cardiomyopathy, Heart failure, Implantable cardioverter defibrillator, Kansas City Cardiomyopathy Questionnaire, Quality of life

## Abstract

**Background:**

Self-reported health status as measured by the Kansas City Cardiomyopathy Questionnaire (KCCQ) in patients with primary prevention implantable cardioverter defibrillators (ICDs) has mainly been reported from randomized trials. However, these studies are often limited to short follow-up and are subject to selection bias. The aim of this study was to assess KCCQ-12 in patients with primary prevention ICD due to either ischemic or nonischemic heart failure.

**Methods:**

This cross-sectional observational study included all patients in Region Gävleborg, Sweden, who because of primary prevention due to heart failure, had an ICD or underwent device replacement between 2007 and 2017. After validation using medical records patients were sent and returned the KCCQ-12 by regular mail.

**Results:**

A total of 118 questionnaires were analyzed (response rate 71.1%). The mean age was 70.9 ± 9.8 years, and a minority was female (n = 20, 16.9%). The mean overall summary score was 71.5 ± 22.4, there was no significant difference between ischemic and nonischemic heart failure (69.5 ± 23.1 vs. 74.4 ± 21.3; *p* = 0.195). Atrial fibrillation at baseline was associated with lower score for the domains Symptom frequency (70.2 ± 23.2 vs. 82.2 ± 19.2; *p* = 0.006) and Social limitation (62.1 ± 26.0 vs. 75.6 ± 26.6; *p* = 0.006) as well as the overall summary score (63.9 ± 21.3 vs. 74.8 ± 22.2; *p* = 0.004).

**Conclusion:**

In a real-world setting, primary prevention ICD patients with heart failure report an acceptable disease-specific health status at long-term follow-up. Ischemic and nonischemic etiology showed similar health status whereas atrial fibrillation was associated with worse outcome.

## Background

An implantable cardioverter defibrillator (ICD) increases survival in those at high risk for sudden cardiac death [1]. An ICD protects from life-threatening arrhythmias by cardioversion, antitachycardia pacing or bradycardia pacing. In combination with a left-ventricular lead it can also offer cardiac resynchronization therapy (CRT-D) for heart failure in selected cases [2]. Guidelines from the European Society of Cardiology (ESC) recommend a primary prophylactic ICD for patients with symptomatic heart failure with New York Heart Association functional classification (NYHA-class) II–III, left ventricular ejection fraction ≤ 35% despite at least three months of optimal medical therapy, and a life expectancy of at least 1 year with good functional status [1].

Patient reported outcome measures through questionnaires have been increasingly used in research settings [3]. In patients with heart failure, the Kansas City Cardiomyopathy Questionnaire (KCCQ) offers a disease-specific measurement of health status and health-related quality of life [4]. The 12-item KCCQ, the KCCQ-12 preserves the psychometric properties of the original questionnaire, shows high correlation with the original scales (> 0.93 for all scales in all clinical settings), high-test–retest reliability (> 0.76 for all domains), and high responsiveness to clinical change [5]. The KCCQ, as well as KCCQ-12 correlate with physician assessed NYHA-class as well as heart failure outcomes [4–6].

The primary aim of this study was to, with a cross-sectional observational design, assess self-reported health status as measured by the KCCQ-12 in an unselected cohort of patients with primary prevention ICD due to ischemic or nonischemic heart failure without tertiary center bias. The secondary aims were to assess whether self-reported health status as measured by the KCCQ-12 were affected by etiology of heart failure (ischemic vs nonischemic), atrial fibrillation before implant, age, sex, complications requiring surgery, and appropriate therapy or inappropriate shock.

## Methods

### Data collection and validity

This cross-sectional observational study included patients from Region Gävleborg in Sweden who, because of primary prevention due to heart failure, had an ICD implanted or who underwent device replacement between 1st January 2007 and 1st of January 2017. Patients were identified from a retrospective observational study. Long-term outcome with regard to appropriate therapy, inappropriate shock, complications requiring surgery, mortality, and cause of death in this cohort has previously been reported [7]. Information about baseline characteristics as well as appropriate therapy, inappropriate shock, complications requiring surgery, and mortality at long-term follow up had been retrieved from electronic medical records (Melior™, Cerner Sverige AB, Stockholm) between March 2017 and February 2018. Eligible patients with primary prevention ICD due to heart failure who had a Swedish postal address were sent and returned a Swedish version of the KCCQ-12 by regular mail during 2019.

### The KCCQ-12

In the KCCQ-12, responses are given on a Likert scale that for each individual item is scored on a scale of 0–100 with higher scores indicating better health. Items are grouped into the four domains; Physical limitation, Symptom frequency, Quality of life, and Social limitation. The score of each domain is calculated as the average of its constituent items. In case of missing values for an item, the score of that item is considered to be the same as the average of the other items in its domain. An overall summary score is calculated as the average of all four domains.

### Statistical analyses

Data were described as frequencies, percentages, and means including standard deviations (±). The t-test was used for comparisons of normally distributed continuous variables. The Mann–Whitney U-test was used for non-normally distributed continuous variables. The Shapiro–Wilk test was used to test for the normality distribution of data. The chi-squared test was used for comparisons of categorical variables. Differences in KCCQ-12 domains and summary scores between groups were tested with Mann–Whitney U-test. The associations between age and KCCQ-12 were tested using Spearman's rank-order correlation. Kruskal–Wallis non-parametric analysis of variance was used to analyze differences between the age strata; 32–59 years, 60–69 years, 70–79 years, and ≥ 80 years. The magnitude of difference in KCCQ-12 domains or summary score between groups was reported as the difference between the means. Two-sided *p* values ≤ 0.05 were considered statistically significant. The software programs Excel 2010 (Microsoft Corporation, Redmond, WA), SPSS version 22 (IBM, Armonk, NY) were used for analyses.

## Results

### Response rate and dropout analysis

**Fig. 1:**
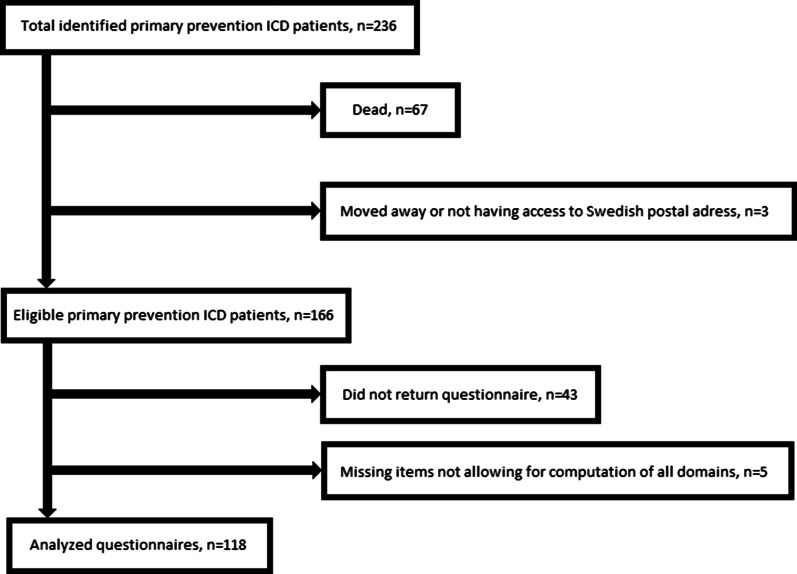
Flow-chart depicting the process for deriving the final sample for analysis

Out of 236 patients identified with primary prevention ICD implantation due to heart failure, 169 were still alive at the time of health status assessment. Out of these 169 patients, 166 had a Swedish postal address and were sent the KCCQ-12 by regular mail including a pre-paid return envelope. The questionnaire was returned by 123 patients, in 118 of these all domains could be calculated. Figure 1 depicts the process for deriving the final sample for analysis. The response rate was 71.1%. The age of those that returned complete questionnaires was similar to those that did not (70.9 ± 9.8 vs. 67.0 ± 12.45 years; *p* = 0.062). The proportion of females was lower among those who returned a complete questionnaire than for those that did not (16.9% vs. 22.9%; *p* < 0.001).

### Cohort characteristics at ICD implantation

The baseline characteristics of the sample (n = 118) at ICD implantation are summarized in Table 1. The mean age at ICD implantation was 65.0 ± 10.4 years. A minority was female (n = 20, 16.9%). The mean age at the time of the survey was similar between females and males (71.2 ± 7.8 vs. 70.9 ± 10.1 years; *p* = 0.904). Almost half of the patients had a CRT-D at first implantation (n = 57, 48.3%). A majority of patients had ischemic etiology (n = 69, 58.5%) and patients with ischemic etiology was older than patients with nonischemic etiology (73.3 ± 8.1 vs. 67.6 ± 10.9; *p* = 0.005). Patients who had atrial fibrillation at baseline were older at the time of the survey than those that did not (74.4 ± 8.1 vs. 69.5 ± 10.1; *p* = 0.013).

**Table 1:** Characteristics at ICD implant of the 118 patients analyzed regarding KCCQ-12

	n (%)
Patients	118
Mean age at implant (years)	65.0 ± 10.4
Mean age at KCCQ (years)	70.9 ± 9.8
Females	20 (16.9)
Ischemic etiology	69 (58.5)
Device type	
ICD-VR	16 (13.6)
ICD-DR	45 (38.1)
CRT-D	57 (48.3)
Hypertension	54 (45.8)
Diabetes mellitus	27 (22.9)
Renal failure*	16 (13.6)
Atrial fibrillation	35 (29.7)
Beta-blockers	106 (89.8)
ACE-i/ARB	111 (94.1)
MRA	75 (63.6)

Data presented as frequencies (percentage in parenthesis)

*ACE-i* angiotensin converting enzyme inhibitor, *ARB* angiotensin receptor blockers, *CRT-D* cardiac resynchronization therapy defibrillator, *ICD-DR* dual lead implantable cardioverter defibrillator, *ICD-VR* single lead implantable cardioverter defibrillator, *KCCQ-12* 12-item Kansas City Cardiomyopathy Questionnaire, *MRA* mineralcorticoid receptor antagonists

*Defined as S-Creatinine ≥ 130 μmol/L

### Cohort characteristics at follow-up

In the final sample, of the 118 patients, the mean time between ICD implantation and follow-up with KCCQ-12 was 5.9 ± 2.3 years. The mean time from ICD implantation to evaluation of baseline characteristics and outcomes such appropriate therapy, inappropriate shock, and complications requiring surgery was 4.1 ± 2.4 years. Out of these 118 patients; 16 (13.6%) experienced appropriate ICD therapy (defined as antitachycardia pacing or cardioversion of ventricular tachyarrhythmia), 5 (4.2%) experienced inappropriate shock (defined as cardioversion in the absence of ventricular tachyarrhythmia), and 16 (13.6%) experienced at least one complication requiring surgical intervention. The mean age was similar for those that experienced appropriate therapy vs those that did not (72.9 ± 8.1 vs. 70.6 ± 10.0 years; *p* = 0.376), for those that experienced inappropriate shock vs those that did not (73.4 ± 8.4 vs. 70.8 ± 9.8; *p* = 0.563), and for those that experienced complications requiring surgery vs those that did not (74.7 ± 8.2 vs. 70.3 ± 9.9; *p* = 0.099).

### Health status measured by KCCQ-12

The results for the four domains and the summary score of the KCCQ-12, for all patients as well as for patients with heart failure of ischemic and nonischemic etiology is given in Table 2. The mean of the overall summary score for all patients was 71.5 ± 22.4. There was no statistically significant difference between ischemic and nonischemic heart failure (69.5 ± 23.1 vs. 74.4 ± 21.3; *p* = 0.195) with regards to the overall summary score, nor was there any statistically significant difference for any domain. In Table 2 the results of the four domains and the summary score are presented for patients with ICD-VR or ICD-DR as compared to patients with CRT-D. The overall summary score was lower in patients with CRT-D compared to ICD-VR/DR (67.7 ± 22.4 vs. 75.1 ± 22.0; *p* = 0.041). Patients with CRT-D reported lower scores in the domain Symptom frequency (74.8 ± 21.2 vs. 82.3 ± 20.5; *p* = 0.040).

**Table 2:** Mean score of the KCCQ-12 in 118 patients with primary prevention ICD due to heart failure

KCCQ-12	All n = 118 Mean score	Ischemic HF n = 69 Mean score	Nonischemic HF n = 49 Mean score	Ischemic versus nonischemic HF *p* value
Physical limitation	68.0 ± 26.4	64.6 ± 26.2	72.8 ± 26.1	0.062
Symptom frequency	78.6 ± 21.1	77.2 ± 21.5	80.6 ± 20.5	0.305
Quality of life	69.7 ± 25.7	68.8 ± 25.7	70.9 ± 25.8	0.582
Social limitation	71.6 ± 27.0	68.8 ± 27.1	75.6 ± 26.7	0.144
Overall summary score	71.5 ± 22.4	69.5 ± 23.1	74.4 ± 21.3	0.195

KCCQ-12	All n = 118 Mean score	ICD-VR/DR n = 61 Mean score	CRT-D n = 57 Mean score	ICD-VR/DR versus CRT-D *p* value
Physical limitation	68.0 ± 26.4	72.0 ± 23.3	63.7 ± 28.9	0.158
Symptom frequency	78.6 ± 21.1	82.3 ± 20.5	74.8 ± 21.2	**0.040**
Quality of life	69.7 ± 25.7	73.2 ± 25.2	66.0 ± 25.9	0.093
Social limitation	71.6 ± 27.0	75.0 ± 27.5	68.0 ± 26.3	0.096
Overall summary score	71.5 ± 22.4	75.1 ± 22.0	67.7 ± 22.4	**0.041**

Statistically significant *p*-values are given in bold

Comparisons are made between ischemic versus nonischemic HF and VR/DR versus CRTD

*CRT-D* cardiac resynchronization therapy defibrillator, *HF* heart failure, *ICD* implantable cardioverter defibrillator, *ICD-VR/DR* single or dual lead implantable cardioverter defibrillator, *KCCQ-12* 12-item Kansas City Cardiomyopathy Questionnaire, *SD* standard deviation

### Age

There was a negative correlation between age at the time of the survey and overall summary score (Spearman’s correlation coefficient, r_S_ = − 0.186; *p* = 0.043), Physical limitation (r_S_ = − 0.230; *p* = 0.012), and Symptom frequency (r_S_ =  − 0.252; *p* = 0.006). But no correlation between age and the Quality of life (*p* = 0.418) or Social limitation (*p* = 0.192) domains.

The KCCQ-12 scores for the age strata; 32–59 years, 60–69 years, 70–79 years, and ≥ 80 years as well as the result of the Kruskal–Wallis tests are shown in Table 3. There was a significant effect of age strata upon Symptom frequency (*p* = 0.046). However, for the youngest age strata, scores for this domain was slightly lower (80.1 ± 22.1) than for the second youngest age strata (84.7 ± 19.7), while after that scores decreased with increasing age with age strata 70–79 years scoring 75.7 ± 19.6 and age strata ≥ 80 years 72.5 ± 23.8. There was no statistically significant difference between the age strata for Physical limitation, Quality of life, or Social limitation.

**Table 3:** KCCQ-12 score in 118 patients with primary prevention ICD due to heart failure stratified by age

KCCQ-12	32–59 years (n = 10)	60–69 years (n = 42)	70–79 years (n = 42)	≥ 80 years (n = 24)	Kruskal–Wallis test
	Mean score	Mean score	Mean score	Mean score	*p* value
Physical limitation	75.8 ± 8.1	71.7 ± 24.9	67.5 ± 26.7	59.0 ± 27.5	0.164
Symptom frequency	80.1 ± 22.1	84.7 ± 19.7	75.7 ± 19.6	72.5 ± 23.8	**0.046**
Quality of life	66.3 ± 28.3	72.9 ± 27.2	67.3 ± 24.4	69.8 ± 25.0	0.520
Social limitation	82.5 ± 22.7	72.0 ± 28.1	70.1 ± 25.1	69.1 ± 30.4	0.564
Overall summary score	75.6 ± 22.6	74.4 ± 22.9	69.6 ± 21.7	68.2 ± 23.0	0.402

Statistically significant *p*-values is given in bold

*ICD* implantable cardioverter defibrillator, *KCCQ-12* 12-item Kansas City Cardiomyopathy Questionnaire

### Sex

There was no significant difference between females and males for the overall summary score (71.1 ± 24.2 vs. 71.6 ± 22.1; *p* = 0.919) or for any of the separate domains; Physical limitation (*p* = 0.679), Symptom frequency (*p* = 0.455), Quality of life (0.858, or Social limitation (*p* = 0.992).

### Subgroup analysis of the KCCQ-12

Subgroup analyzes for atrial fibrillation, appropriate therapy, inappropriate shock, and complications requiring surgery are shown in Table 4. Patients with a history of atrial fibrillation before ICD implant had at follow-up significantly lower score for the domains Symptom frequency (70.2 ± 23.2 vs. 82.2 ± 19.2; *p* = 0.006) and Social limitation (62.1 ± 26.0 vs. 75.6 ± 26.6; *p* = 0.006) as well as the overall summary score (63.9 ± 21.3 vs. 74.8 ± 22.2; *p* = 0.004), but there was no statistically significant difference in the scores for the domain Quality of life (*p* = 0.119). However, for the domain Physical limitation there was a trend toward significance (*p* = 0.051) with atrial fibrillation patients having lower scores (61.0 ± 27.3 vs. 70.9 ± 25.6). Patients with and without a history of appropriate therapy or complications requiring surgery had similar scores on all KCCQ-12 domains and the overall summary score. There were few patients who had received inappropriate therapy (n = 5). Patients who had received inappropriate therapy had significantly lower scores for both the domain Social limitation (43.3 ± 19.0 vs. 72.5 ± 22.1; *p* = 0.015) and the overall summary score (50.7 ± 19.0 vs. 72.5 ± 22.1; *p* = 0.031), but there was no statistically significant differences in the domains Physical limitation, Symptom frequency, or Quality of life.

**Table 4:** Subgroup analyses of KCCQ-12 scores in 118 patients with primary prevention ICD due to heart failure

KCCQ-12	Atrial fibrillation n = 35		Appropriate therapy n = 16		Inappropriate shock n = 5		Complications n = 16	
	Mean difference	*p* value	Mean difference	*p* value	Mean difference	*p* value	Mean difference	*p* value
Physical limitation	− 10.0	0.051	9.9	0.238	− 17.0	0.187	4.5	0.782
Symptom frequency	− 12.0	**0.006**	2.4	0.946	− 18.6	0.098	− 0.4	0.864
Quality of life	− 8.2	0.119	0.7	0.795	− 18.0	0.168	2.5	0.898
Social limitation	− 13.5	**0.006**	3.9	0.958	− 29.6	**0.015**	1.5	0.930
Overall summary score	− 10.9	**0.004**	3.6	0.841	− 21.8	**0.031**	2.0	0.925

Statistically significant *p*-values are given in bold

Positive mean difference indicates higher values

*ICD* implantable cardioverter defibrillator, *KCCQ-12* 12-item Kansas City Cardiomyopathy Questionnaire

## Discussion

In real-world primary prevention ICD cohorts, KCCQ-12 scores might be lower than in cohorts reported from randomized trials. However, KCCQ-12 scores in this population considering the long-term follow-up appear to be generally acceptable, indicating selection of patients with high functional status at implantation. A change of at least 5 points in KCCQ-12 scores is usually considered to be the minimal clinically important difference [5, 8].

The short form of the KCCQ, the KCCQ-12 was created as a tool for easier implementation into clinical practice and preserves the psychometric properties of the original KCCQ [5]. For this reason, the KCCQ-12 was used in this study, however this makes comparisons to some studies more difficult. In the Multicenter Automatic Defibrillator Implantation Trial With Cardiac Resynchronization Therapy (MADIT) trial, health status was measured with the KCCQ, due to reporting of all separate domains, an approximation of the KCCQ-12 overall summary score can be calculated [9]. At inclusion the mean of the overall summary score for the 675 patients with only ICD was 75.2 for all KCCQ domains and 75.1 for all KCCQ-12 domains, for the 1024 patients with CRT-D the mean of the overall summary score was 75.6 for all KCCQ domains and 75.4 for all KCCQ-12 domains [9]. This demonstrates how similar the overall summary score is for KCCQ and KCCQ-12, in a population with primary prevention ICD due to heart failure, the removal of the domains symptom stability and symptom burden does not affect the overall summary score.

The MADIT included patients with left ventricular ejection fraction ≤ 30% but only NYHA class I–II. At follow-up after three years, the mean KCCQ overall summary score for left bundle branch block patients solely with ICD was 80.0 and for patients with CRT-D was 83.4 [9]. Both higher than the mean KCCQ-12 overall summary score of 71.5 in our cohort after a mean follow-up of 5.9 years. This is partly explained by the inclusion of only NYHA class I–II in the MADIT while primary prevention ICD guidelines recommend implantation of an ICD for NYHA II–III [1]. In the MADIT patients were randomized to the intervention of CRT-D implantation and CRT-D was shown to increase KCCQ scores. In our study, patients where not randomized to CRT-D. In real world cohorts patients with more severe symptoms might be more prone to receive CRT-D. This likely explains why patients with CRT-D had lower KCCQ scores in our cohort.

In the Danish Study to Assess the Efficacy of Implantable Cardioverter-Defibrillators (DANISH) trial heart failure patients were randomized to either ICD or usual clinical care and health status was measured using the Minnesota Living with Heart Failure Questionnaire (MLHFQ) [10]. After 32 months the change in mean MLHFQ overall score was similar between the ICD and control group (*p* = 0.82). Probably differences between heart failure patients with and without primary prevention ICD are primarily not caused by ICD implantation but rather by the selection process.

### Age

There were moderate correlations between age and the KCCQ-12 domains Physical limitation, Symptom frequency, and the overall summary score. This was strongest for Symptom frequency (rS =  − 0.252), that also was the domain that showed significant differences between the prespecified age strata. It is likely that Quality of life and Social limitation, domains that did not show statistically significant correlations with age, reflects more of a subjective interpretation of health-related quality of life, while domains such as Physical limitation and Symptom frequency reflect more of an objective assessment of heart failure specific health status that is more age-dependent. It is also likely that physicians offer all younger patients that fulfill guideline criteria a primary prevention ICD, while for older patients there is a selection, where those patients who have higher health-related quality of life are more likely to have an ICD implanted. A large study of outpatient heart failure patients showed a small negative effect (mean difference − 5.5 per 10-year increment, adjusted for sex and ethnicity) of age upon KCCQ overall summary score for those patients who are ≥ 70 years while this was not seen for patients < 70 years of age [11].

### Sex

The proportion of females was notably low (16.9%), this was partly due to females in our cohort being somewhat less likely to return the questionnaire as was seen in the drop-out analysis with chi-squared test (*p* < 0.001), but also due to a low proportion of females among eligible participants for the study in the cohort of primary prevention ICD patients (22.9%). The low proportion of females in primary prevention ICD cohorts is well-known, in one study with pooled data from eleven European national registries the proportion of females was 18.7% [12]. In our study sex category had no effect on any of the KCCQ-12 domains or overall summary score. In heart failure, differences between the sexes in health-related quality of life and health status are controversial. While results of many studies have been heterogeneous, some have reported lower KCCQ scores in female than in male heart failure patients, independently from other clinical factors [11, 13]. The reason for lower proportions of women in ICD cohorts is unclear, females with primary prevention ICD has been shown to have a lower mortality and lower risk of appropriate therapy than males [12]. Therefore, sex differences in heart failure health status or health-related quality of life, if any exist, might differ between general heart failure populations and primary prevention ICD cohorts.

### Appropriate therapy

Previous prospective observational studies have identified ICD shocks as a factor decreasing health-related quality of life and physical activity as well as increasing anxiety [14]. In our study no significant association between appropriate therapy and health status measured by the KCCQ-12 was seen. In the PainFree SST clinical trial, anxiety remained increased for the 24 month follow-up after a shock, reduction in daily activity was seen but returned to normal after 3 months [14]. Our patients with a longer time with an ICD (5.9 ± 2.3 years) might reflect a population in which health status no longer is affected by previous ICD therapy. However, the lack of a statistically significant association between appropriate therapy and health status might also be due to our lower sample size resulting in a type II error.

### Inappropriate shock

Inappropriate therapy reached the significance level for the Social limitation domain and the overall summary score. While it was clear that this subgroup in our cohort scored generally worse (difference in mean overall summary score − 21.8), the number of patients in this subgroup was small (n = 5). Due to this, the magnitude of decrease in KCCQ-12 score is uncertain. However, several mechanisms that could lead to lower health status in these patients exist, such as anxiety after painful shocks or them serving as a marker of rapid atrial fibrillation, the most common reason for inappropriate shocks [15]. It has been shown that inappropriate shocks due to atrial fibrillation, but not due to lead failure, are associated with increased all-cause mortality [15].

### Atrial fibrillation

In our cohort, a history of atrial fibrillation before ICD implant predicted significantly lower KCCQ-12 scores at follow-up (difference in mean overall summary score − 10.9). There was a statistically significant reduction of similar size in both Symptom frequency and Social limitation, as well as a trend toward the same in other domains. Atrial fibrillation is associated with marked decreases in health-related quality of life [16]. In patients with heart failure and concomitant atrial fibrillation, therapy with catheter ablation improves health-related quality of life as well as heart failure specific health status measured by the MLHFQ [17]. The association between atrial fibrillation and lower health status as measured by the KCCQ-12 in heart failure patients could be due to atrial fibrillation being more common in severe heart failure, atrial fibrillation leading to a deterioration of left ventricular systolic function over time, or atrial fibrillation and heart failure both contributing to the burden of disease in ways that the KCCQ-12 cannot discriminate between [18].

### Complications requiring surgery

There were no statistical differences in any domain or in the KCCQ-12 overall summary score. Lead-related problems are the most common complications that requires new surgery, after 10 years 25% of leads have been affected by complications [19]. In our whole ICD cohort, lead dislodgement or lead dysfunction constituted 60% of complications requiring surgery [7]. Therefore, while KCCQ-12 scores predict several outcomes, it is likely that lead-related complications depend more on lead-related factors rather than health status. In one study on patients with an ICD and hypertrophic cardiomyopathy, complications requiring surgery did not affect health-related quality of life measured by the SF-36 [20]. However, in that study as well as in the current study, follow-up time was long, it is plausible that complications requiring surgery would affect health status in the short term. This should be evaluated in future trials.

### Strengths and weaknesses

This cross-sectional observational study provides real-world data regarding KCCQ-12 scores in an unselected cohort of patients with primary prevention ICD due to heart failure with long-term follow-up. All patients and variables have been validated from electronic medical records by physicians. The total number of patients was relatively low making interpretation more difficult. Few patients in some subgroups make some of the subgroup analyses prone to type I and II error. While the response rate of 71% was considered acceptable, 29% of patients did not return the questionnaire thus there is a risk of selection bias. Our study shares the limitation of a low proportion of females seen in numerous ICD cohorts. It is unknown to which extent geographical differences in the ICD management within a country matters and if interpretations can be generalized to other countries.

### Clinical perspective

This observational pragmatic study demonstrates a patient-reported health status which is lower than in some prospective studies including randomized controlled trials. Still, this study indicates an acceptable health status in long-term survivors after ICD-implant. The selection of ICD candidates seems to be adequate as the health status likely can be translated into moderate burden of symptoms. Nevertheless, this study highlights the importance of optimal medical management. Therefore, modern optimal heart failure medication and structured clinical care is warranted in addition to device follow-up.

## Conclusions

In a real-world pragmatic setting, primary prevention ICD patients with heart failure report an acceptable disease-specific health status according to KCCQ-12 at long-term follow-up. Ischemic and nonischemic underlying etiology showed similar health status whereas atrial fibrillation was associated with worse outcome.

## Data Availability

Upon request.

## Abbreviations

CRT-D: Cardiac resynchronization therapy defibrillator
ESC: European Society of Cardiology
ICD: Implantable cardioverter defibrillator
KCCQ: Kansas City Cardiomyopathy Questionnaire
KCCQ-12: 12-Item Kansas City Cardiomyopathy Questionnaire
MLHFQ: Minnesota Living with Heart Failure Questionnaire
NYHA class: New York Heart Association functional classification
